# Exposure of Smaller and Oxidized Graphene on Polyurethane Surface Improves its Antimicrobial Performance

**DOI:** 10.3390/nano10020349

**Published:** 2020-02-18

**Authors:** Inês Borges, Patrícia C. Henriques, Rita N. Gomes, Artur M. Pinto, Manuel Pestana, Fernão D. Magalhães, Inês C. Gonçalves

**Affiliations:** 1 i3S—Instituto de Investigação e Inovação em Saúde, Universidade do Porto, Rua Alfredo Allen, 208, 4200-135 Porto, Portugal; ines.s.borges25@gmail.com (I.B.); ap.henriques@ineb.up.pt (P.C.H.); argomes@i3s.up.pt (R.N.G.); mvasconcelos@chsj.min-saude.pt (M.P.); 2 INEB—Instituto de Engenharia Biomédica, Universidade do Porto, Rua Alfredo Allen, 208, 4200-135 Porto, Portugal; 3 FEUP—Faculdade de Engenharia, Departamento de Engenharia Metalúrgica e de Materiais, Universidade do Porto, Rua Alfredo Allen, 208, 4200-135 Porto, Portugal; 4 LEPABE, Faculdade de Engenharia, Universidade do Porto, Rua Dr. Roberto Frias, 4200-465 Porto, Portugal; fdmagalh@fe.up.pt; 5 Department of Nephrology, São João Hospital Center, EPE, Alameda Prof. Hernâni Monteiro, 4200-319 Porto, Portugal; 6 Department of Medicine, Faculty of Medicine, University of Porto, Alameda Prof. Hernâni Monteiro, 4200-319 Porto, Portugal

**Keywords:** biomaterials, catheter-related infections, bacterial adhesion, composites, coatings

## Abstract

Catheter-related infections are a common worldwide health problem, highlighting the need for antimicrobial catheters. Here, antibacterial potential of graphene nanoplatelets (GNP) incorporated in the commonly used polymer for catheter manufacture—polyurethane (PU)—is investigated. Two strategies are explored: melt-blending, producing a composite, and dip coating, where a composite layer is deposited on top of PU. GNP with different lateral sizes and oxidation degrees—GNP-M5, GNP-M15, GNP-M5ox, GNP-M15ox—are applied in both strategies, and the antimicrobial potential towards *Staphylococcus epidermidis* of GNP dispersions and GNP-containing PU evaluated. As dispersions, oxidized and smaller GNP powders (GNP-M5ox) inhibit 74% bacteria growth at 128 µg/mL. As surfaces, GNP exposure strongly impacts their antimicrobial profile: GNP absence at the surface of composites yields no significant effects on bacteria, while by varying GNP: PU ratio and GNP concentration, coatings enhance GNP exposure, depicting an antimicrobial profile. Oxidized GNP-containing coatings induce higher antibacterial effect than non-oxidized forms, particularly with smaller GNPox, where a homogeneous layer of fused platelets is formed on PU, leading to 70% reduction in bacterial adhesion and 70% bacterial death. This pioneering work unravels how to turn a polymer clinically used to produce catheters into an antimicrobial surface, crucial to reducing risk of infection associated with catheterization.

## 1. Introduction

Catheters are medical devices routinely used in patients in different healthcare centers [1]; catheter-related infections (CRI) are however a recurrent and threatening problem, especially if progressing to bloodstream infection, the third leading cause of hospital-acquired infections. These can cause morbidity, prolonged hospitalizations and ultimately death [2], being associated with elevated medical costs and therefore representing a serious worldwide health problem [3,4]. CRI are mainly caused by colonization of the catheter hub or tubing by microorganisms from the patient’s skin or external sources—*Staphylococcus epidermidis* being the major responsible one [5]—but also by contamination of the catheter lumen by circulating bacteria [3,6,7]. Treatment involves catheter replacement or removal, and antibiotic therapy—inefficient in most cases of biofilm formation [3] and associated with bacterial resistance [7]. To overcome and prevent CRI, apart from better clinical practices during catheter insertion/manipulation, new technologies and products have been continuously applied in the clinics, namely impregnation of catheters and dressings with antiseptics or antibiotics [3,4,8]. Other strategies are being investigated, focusing on conferring anti-adhesive and/or bactericidal properties through surface modification of the medical-grade polyurethane (PU)—a thermoplastic elastomer from which most catheters are made of [9,10]. Strategies include physical and chemical modifications such as incorporation or coatings with antibiotics or antiseptics [11,12,13], topography modification with micropatterns [14], functionalization or coating with antimicrobial peptides (AMPs) [15] and antimicrobial nanomaterials, such as silver nanoparticles (Ag NPs) [16], titanium oxide (TiO_2_) NPs [17], carbon nanotubes (CNT) [18], bioactive compounds [19], and graphene oxide (GO) [20]. Approaches that depend on eluting bactericidal agents have many drawbacks, namely the limitation of the amount loaded, difficulty in controlling the release rate and the concentration of the released agents during the application period [21,22]. In particular, antiseptics and antibiotics can induce bacteria resistance as a consequence of their prolonged use [23] and loose efficiency along time due to leaching [8]. Therefore, permanent modification of the catheter surface with antimicrobial properties is the ideal solution, aiming preventive strategies that act at the frontline by avoiding bacteria adhesion, colonization and consequent biofilm formation.

Graphene-based materials (GBM), a new family of nanomaterials, have attracted a lot of attention in the scientific community, with increasing interest and potential in biomedical applications [24,25,26,27,28]. The antibacterial effect of these materials was first explored in suspension in 2010 by Hu et al. [29] and since then increasing number of studies have described GBM as having antibacterial activity, making them strong candidates for antimicrobial applications [30,31,32]. Antimicrobial properties of these materials have been addressed in a large number of reviews [33,34,35,36,37,38,39], but mostly in suspension; their antimicrobial performance is however different when incorporated in surfaces [40,41]. The mechanisms of action have been thoroughly explored, with recently published reviews covering the most important conclusions so far. As suspensions, GBM act either through wrapping, isolating bacteria from the environment, or through insertion or nano knife, physically piercing the membrane, although oxidative stress can also be stimulated [31,42]. As surfaces, they act after direct contact with bacteria, with predominant mechanisms depending on the exposure of either basal planes or sharp edges: electron transference and oxidative stress induction occur mostly when only basal planes are exposed, while besides these, physical damage of the membrane (due to insertion or protein-protein destabilization) can also occur when both structures contact bacteria [40,43,44]. Very few studies report the use of GBM to confer antibacterial properties to PU [45,46,47]: An et al. produced polylactic acid (PLA)/PU composite films reinforced with GO with antibacterial activity against both *Staphylococcus aureus* and *Escherichia coli* [45]; in another work, hyperbranched polyurethane/reduced GO decorated with sulphur nanoparticles (HPU/SRGO) composite showed microbial inhibitory effects against *S. aureus*, *E. coli* and *Candida albicans*, inhibiting the growth rate and causing membrane disruption [46]; in the study of Tang et al., PU composites incorporated with GO and with slightly reduced GO modified with polyethylenimine (SRGO-PEI) showed an antiadhesive effect towards *E. coli* [47]. However, most of the times, either PU or GBM are combined with other polymers or materials, preventing the understanding of their effective role on the antibacterial performance; GBM type and oxidation state as well as the influence of the method of incorporation and degree of GBM exposure at the surface are seldom evaluated.

This work aims to incorporate graphene nanoplatelets—GNP, composed of 2 to 10 graphene sheets—into PU through two different methods compatible with catheter fabrication process—melt-blending and dip coating—and investigate the antimicrobial potential of the obtained surfaces for application on PU catheters aiming to prevent catheter-related infections. The antibacterial potential towards *S. epidermidis* of GNP powders with different lateral size and oxidation degree—GNP-M5 (5 µm), GNP-M15 (15 µm) and oxidized forms, GNP-M5ox, GNP-M15ox—was initially assessed and the impact of these parameters on GNP exposure and antimicrobial potential of the obtained surfaces was investigated. Since in GNP-related literature, evaluation fails in the complete assessment of their antibacterial properties and adaptability to different mechanisms of antimicrobial surface design (antimicrobial-releasing, contact-killing and non-adhesiveness) [48], in our study bacteria viability and quantification were conducted, both in the supernatant and adherent to the surface, by adaptation of ISO 22196. 

## 2. Materials and Methods 

### 2.1. Oxidation of Graphene Nanoplatelets (GNP)

Graphene nanoplatelets of grade M (xGnP^®^ Graphene Nanoplatelets Grade M) with 5 μm (GNP-M5) and 15 μm of diameter (GNP-M15) were purchased from XG Sciences (Lansing, USA). Oxidized forms of GNP-M5 and GNP-M15, designated GNP-M5ox and GNP-M15ox, were produced by a modified Hummers’ method (MHM) using a GNP-M:KMnO_4_ mass ratio of 1:6. 4 g of GNP were added to a mixture of 160 mL of 95%–97% H_2_SO_4_ (VWR, Darmstadt, Germany) and 40 mL of H_3_PO_4_ (VWR, Germany) [49,50,51]. Oxidized GNP (GNPox) was repeatedly washed by resuspending in distilled water (*d*H_2_O) and centrifuging until the pH of the decanted washing water was close to the one of *d*H_2_O. To obtain dried GNPox for composite production, freeze-drying was performed at a temperature of −80 °C and at a pressure of 0.008 mBar for three days.

### 2.2. Production of GNP-Containing Polyurethane 

GNP-containing polyurethane surfaces were produced using two approaches: melt-blending, originating PU/GNP composites that were composed of PU with GNP as nanofiller, and dip coating, yielding PU/GNP coatings, where a thin composite layer of GNP dispersed in PU was deposited on top of PU films.

#### 2.2.1. Production of PU/GNP Composites 

Pellets of Tecothane TT-1095A medical grade polyurethane (PU) were purchased from Lubrizol LifeSciences (Ohio, USA). PU composites with non-oxidized (GNP-M5 and GNP-M15) or oxidized (GNP-M5ox and GNP-M15ox) GNP powders were produced by melt-blending in a Micro-Compounder Xplore (DSM) MC5 (5 mL capacity, vertical, co-rotating twin-screws) at 200 °C and mixing for 3 min with a screw speed of 50 rpm, followed by injection molding performed using the Xplore micro injection moulder IM5.5. (Xplore Instruments BV, Sittard, The Netherlands) Different GNP contents were tested (0.5, 1, 5 and 10 wt%) and dog bone shaped composites obtained (Figure S1).

#### 2.2.2. Production of PU/GNP Coatings

a. PU Films

PU films were prepared by solvent casting [52]. Briefly, PU pellets were dissolved at 12.5% *w*/*v* in tetrahydrofuran (THF, JMGS, Odivelas, Portugal), and 80 mL of this solution were casted into clean glass petri dishes of 14 cm of diameter. Petri dishes were covered with aluminum foil and left at room temperature (RT) for 48 h for slow evaporation of THF; films were removed and cut into squares of 2 × 2 cm^2^.

b. PU/GNP coatings on PU films

Coating formulations containing PU and GNP were prepared by solvent mixing. Briefly, GNP dispersions were prepared in dimethylformamide (DMF, Fisher Scientific, UK) at different concentrations (0.25, 0.5 and 1 mg/mL) with mechanical stirring and three periods of 1.5 min ultrasonication using a Hielsher UIP 1000 probe (1000 W, 20 kHz). Then, PU solution in DMF (50 mg/mL) was added to the GNP dispersions in different GNP to PU weight proportions (1:1, 1:0.5 and 1:0.25—diminishing the quantity of PU in the coating formulation), under continuous stirring followed by three periods of 1.5 min ultrasonication, to obtain homogenous PU/GNP dispersions in DMF. PU films were immersed vertically in the coating formulations for 30 s and dried vertically at 60 °C for 12 h in a vacuum oven.

### 2.3. Characterization of GNP Powders and GNP-Containing Polyurethane

#### 2.3.1. Transmission Electron Microscopy (TEM)

Size of GNP powders was confirmed based on images obtained from TEM using a JEOL JEM 1400 TEM (Tokyo, Japan) coupled to a digital camera CCD Orious 1100 W (Tokyo, Japan) at the Histology and Electron Microscopy Service (HEMS, i3S, Porto, Portugal). For that, a small amount of dry GBMs were dispersed in water and dropped on 200 mesh copper TEM grids.

#### 2.3.2. Scanning Electron Microscopy (SEM)

Morphology of GNP powders and GNP-containing polyurethane—PU/GNP composites and PU/GNP coatings—was observed using a high-resolution SEM FEI Quanta 400FEG, with acceleration voltage of 10 or 15 kV. Powder samples and PU/GNP coatings were placed directly on conductive carbon tape. Composites were fractured transversely after freezing with liquid nitrogen, placed on carbon tape and both the surface and the transversal fracture surface were analyzed. To improve surface conductivity, all samples were sputtered with Au/Pd using a SPI Module Sputter Coater equipment for 50 s using a 15 mA current. 

#### 2.3.3. X-ray Photoelectron spectroscopy (XPS)

Chemical composition and oxidation degree of GNP powders and selected PU/GNP coatings were determined by XPS analysis. Measurements were performed using a Kratos Axis Ultra HSA equipment with a monochromatic Al X-ray source operating at 15 kV (90 W). The analyzer pass energy was 80 eV for survey spectra and 40 eV for C 1s high-resolution spectra. Analyzed scanning area was close to 5 mm^2^ and photoelectron take-off angle was 90° for powders and 70° for coating analysis. The effect of the electric charge was corrected by setting the reference of the C 1s peak to 284.6 eV. Spectra deconvolution was performed with CasaXPS processing software (version 2.3.16), using background type Shirley and Gaussian–Lorentzian (70:30) function to fit all peaks with exception of sp^2^ carbon peak, fitted using an asymmetric Lorentzian function with asymmetry parameter of 0.14 [53]; peak positions were established based on literature data [54].

#### 2.3.4. X-ray Diffraction (XRD)

Oxidation degree, thickness, interlayer spacing between sheets, and crystallinity of GNP powders were determined by XRD analysis. Measurements were performed in a SmartLab Rigaku^®^ diffractometer (Rigaku, Tokyo, Japan) that operates with 45 kV and 200 mA and has a Cu-K*α* radiation source (wavelength of 1.540593 Å) in a Bragg-Brentano configuration. Samples were measured in a rotative system (30 deg min^−1^), in the range of 5–50 theta (2*θ*), with a step of 0.01°. Spectra were baseline corrected.

#### 2.3.5. Raman Spectroscopy

Presence of GNP on the surface of selected PU/GNP coatings was evaluated by Raman spectroscopy. Spectra were acquired using a confocal Raman microscope, LabRAM HR800 UV, Horiba Jobin-Yvon, with a laser excitation of 633.3 nm and acquisition time of 20 sec in a depth of 50 µm. A 100× objective (Olympus) lens was used. Between two and four spectra (~19 µm^2^ each) were acquired for each sample, followed by baseline correction, and results are presented as mean of the spectra.

#### 2.3.6. Water Contact Angle 

Wettability of GNP-containing polyurethane was evaluated by water contact angle measurements using the dynamic sessile drop method and performed with an OCA 15 goniometer equipped with a video CCD camera and SCA 20 software (DataPhysics, Filderstadt, Germany). Samples were placed in a closed thermostatic chamber (25 °C) saturated with *d*H_2_O to prevent droplet evaporation. Drops of 4 µL of *d*H_2_O were placed on samples surface and images were taken 30 times/min during 2 min. Droplet profiles were fitted using Young-Laplace mathematical function. Water contact angles were calculated by extrapolating the time-dependent curve to zero. Results are the average of six measurements on six independent samples. 

#### 2.3.7. Rubbing Test

GNP adhesion of the PU/GNP coatings to the PU films was determined by rubbing a white eraser on samples surface with constant pressure. The resultant shade of grey on the rubber surface was used as a qualitative indication of GNP detachment.

### 2.4. Antibacterial Activity Assays

#### 2.4.1. Bacterial Strains and Growth Conditions 

*Staphylococcus epidermidis* (*S. epidermidis*, ATCC 35984), clinically isolated from catheter sepsis, was obtained from the American Type Culture Collection. Bacteria were grown in Trypticase Soy Agar (TSA, Merck, Darmstadt, Germany) plates overnight at 37 °C and used immediately or stored at 4 °C up to one week. Two colonies were collected, inoculated into 5 mL of Trypticase Soy Broth (TSB, Merck) and cultured overnight at 37 °C under stirring at 150 rpm. 

#### 2.4.2. Antibacterial Activity of GNP Dispersions

Overnight culture of *S. epidermidis* was adjusted to 2 × 10^5^ Colony-Forming Units (CFUs)/mL in fresh TSB medium. GNP-M5 and GNP-M15 powders and respective oxidized forms (GNP-M5ox and GNP-M15ox) were dispersed in *d*H_2_O using ultrasound bath; 2-fold serial dilutions were prepared with concentrations ranging from 2 to 1024 μg/mL. 100 µL of each of the different dilutions were added to 100 µL of the initial bacterial inoculum, obtaining a final bacteria concentration of 1 × 10^5^ CFU/mL and GNP concentration ranging from 1 to 512 μg/mL. Incubation was performed for 18–24 h at 37 °C under static conditions in a 96-well plate. Controls of TSB, inoculated TSB and materials suspensions in TSB (without bacteria) were performed. To avoid water evaporation, empty wells of the 96-well plate were filled with *d*H_2_O and the plate was placed in a container with moistened paper. The determination of the minimal inhibitory concentration (MIC) and the minimum bactericidal concentration (MBC) is commonly performed by visual analysis of bacterial growth inhibition on the bottom of the wells and by plating the content of the first three wells where visible growth is not observed, respectively [55]. However, since the presence of GNP turns the dispersions black, visual assessment of bacterial growth was not possible in this case. As such, for MIC determination, the metabolic activity of bacteria was evaluated by Alamar blue assay. For that, 20 µL of resazurin were added to each well and incubated for 3 h at 37 °C. Then, 100 µL were transferred to a 96-well black plate and the relative fluorescence units (RFUs) (*λ_ex_*: 530 nm and *λ_em_*: 590 nm) were measured in a microplate fluorometer (Spectra Max GeminiXS, Molecular Devices). Wells where reduced metabolic activity was detected, and therefore considered MICs, were selected for MBC evaluation. This was performed by CFUs counting, where viable bacteria are counted by the agar plate culture method. For that 10-fold serial dilutions were made and three drops of 10 µL per dilution were plated in TSA and incubated overnight at 37 °C. Finally, the CFUs were counted for cell viability evaluation.

#### 2.4.3. Antibacterial Activity of GNP-Containing Polyurethane

PU/GNP composites were cut into squares of 1 × 1 cm^2^ and PU/GNP-coated films were cut into disks of 14 mm diameter. All samples were rinsed 3 times with *d*H_2_O to remove possible impurities resultant from the cutting process, dried with argon (Ar) and sterilized using ethylene oxide. 

The antibacterial activity of the surfaces was assessed following an adaptation of the standard “ISO 22196—Measurement of antibacterial activity on plastics and other nonporous surfaces”. Overnight culture of *S. epidermidis* was adjusted to a target concentration of 6 × 10^5^ CFUs/mL in fresh TSB medium. A drop of 15 μL bacterial suspension was placed on top of surfaces and a 9 mm diameter polypropylene (PP) sterile film was placed on top to promote contact between bacteria and the sample surface. Empty wells were filled with *d*H_2_O and plates placed inside a container with moistened paper to avoid water evaporation. Samples were incubated for 24 h under static conditions at 37 °C. PU controls were used to measure viable cells immediately after inoculation to determine the recovery rate of bacteria from the test specimens. Experiments were conducted with five replicates of each sample and replicates of each sample were incubated with only TSB medium as contamination controls. After incubation, samples were rinsed with 1.5 mL of fresh TSB to detach the PP film and loosely adherent bacteria. The supernatant was collected to assess planktonic bacteria and the samples surfaces were analyzed regarding adherent bacteria. 

a. Adherent Bacteria

Bacteria adherent to the surfaces were assessed by staining total and dead bacteria through DNA staining. Samples were rinsed twice with sterile phosphate-buffered saline (PBS), and for total bacteria staining the composites were incubated with 5 μg/mL Hoechst 33342 (Molecular Probes, Oregon, USA) at 37 °C and coatings with 5 μM Draq5 (BioStatus, Leicestershire, UK) at RT for 15 min and protected from light, due to autofluorescence issues of PU. Samples were then incubated with 1.25 μg/mL propidium iodide (PI) (Molecular Probes, Oregon, USA) at RT and protected from light, which only penetrates bacteria with damaged membranes (dead), staining them red. Afterwards, samples were rinsed with PBS and transferred to an uncoated black 24-well μ-plate (IBIDI, Munich, Germany) with the surface facing the bottom. Images of nine fields per sample were acquired using the high-content screening microscope IN Cell Analyzer 2000 (GE Healthcare Life Sciences, Illinois, USA) in the DAPI (455 nm), Cy5 (705 nm) and Cy3 (605 nm) channels with a Nikon 40x/0.95 NA Plan Fluor objective. Quantification of bacteria on the images was performed using the ImageJ software.

b. Planktonic Bacteria

Metabolic activity of planktonic bacteria was evaluated by Alamar blue assay. Resazurin was added to supernatants (10% *v*/*v*) and incubated for 1 h at 37 °C. RFUs were measured (λ_ex_: 530 nm; λ_em_: 590 nm) in a microplate fluorometer (Spectra Max GeminiXS, Molecular Devices, California, USA) and correlated to the metabolic activity of bacteria present in the medium. Viable cell count was also performed using the agar plate culture method. For that, serial dilutions (10^−2^, 10^−4^, 10^−6^) of the supernatants were prepared and three drops of 10 μL of each dilution plated in TSA. Plates were incubated overnight at 37 °C and CFUs counted. 

## 3. Results and Discussion

This work explores the antimicrobial potential of graphene-based materials (GBM), in particular graphene nanoplatelets (GNP), for application on polyurethane (PU) catheters in order to prevent catheter-related infections. To our knowledge, modification of PU alone using GNP for antimicrobial applications has never been reported before, even though the two have been combined for other applications such as the improvement of mechanical, thermal and electrical properties [56,57,58,59,60,61,62,63]. Therefore, it was important to test different types of GNP by varying the platelet size and oxidation and, moreover, test different incorporation strategies such as the production of composites and the application as a coating. For this, four distinct types of GNP were explored, namely commercial non-oxidized GNP-M5 and GNP-M15, with two different lateral sizes (5 and 15 µm), and their respective oxidized forms, GNP-M5ox and GNP-M15ox, produced by chemical oxidation through the modified Hummers method.

### 3.1. GNP Powders

**Figure 1:**
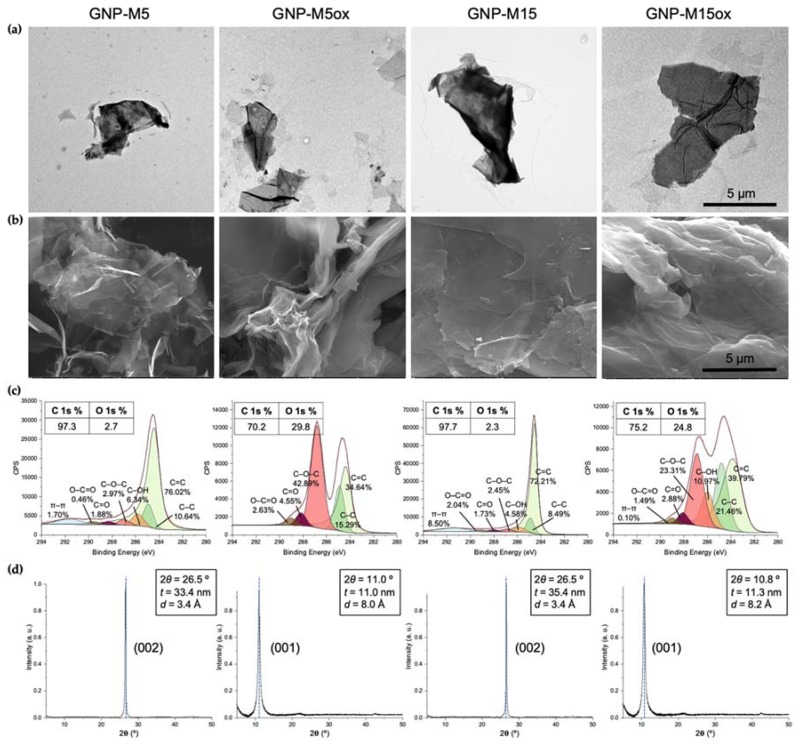
Characterization of graphene nanoplatelet (GNP) powders (GNP-M5, GNP-M5ox, GNP-M15 and GNP-M15ox): (**a**) TEM images (first row) and (**b**) SEM (second row) images (scale bar = 5 µm), (**c**) XPS atomic composition and carbon high-resolution spectra with respective functional groups, and (**d**) XRD patterns with diffraction angle (2*θ*), thickness (*t*) and interlayer spacing between sheets (*d*).

GNP powders were morphologically and chemically characterized before assessing their antibacterial potential. Difference between platelets lateral size was confirmed by TEM; both TEM and SEM demonstrated differences in their structure and morphology, with platelets morphology being closely related to their oxidation state (Figure 1a,b). Non-oxidized GNP-M5 and GNP-M15 powders presented a planar conformation, flat and smooth surfaces with irregular and sharp edges, as well as a more compact structure—denoted by the darker areas in the TEM images. Platelets were closely associated with each other due to inter-platelets hydrogen bonds and hydrophobic interactions that lead to a higher aggregation and agglomeration [64]. Oxidized GNP powders lost the planar conformation, with flakes appearing highly wrinkled, with folded sharp edges. This can occur due to formation of intra-platelet hydrogen bonds through the oxygen-containing functional groups present at the platelets’ edges and basal planes [49]. XPS analysis confirmed GNP oxidation and chemical composition (Figure 1c): non-oxidized GNP presented low oxygen content, with atomic percentage of O 1s being lower than 3%—this small amount of oxygen is associated with GNP preparation process that consists in graphite intercalation and thermal exfoliation, leading to addition of small amounts of oxygen groups mostly at the platelets edges [49]. GNP oxidation increased the oxygen content up to 30%, associated with intercalation of oxygen-containing groups and water molecules. The oxidation process led to a decrease in the sp^2^ carbon bonds, with reduction of the C=C groups from ~70% to ~30%, leading to a decrease of the sp^2^/sp^3^ ratio from 7.1 in GNP-M5 to 2.3 in GNP-M5ox and from 8.5 in GNP-M15 to 1.9 in GNP-M15ox. Predominant oxygen-containing groups in GNP-M5ox are epoxy groups (C-O-C), followed by carbonyl (C=O) and carboxyl (O-C=O) groups, while GNP-M15ox also shows the presence of hydroxyl (C-OH) groups. Oxidation is corroborated by the XRD patterns of GNP powders (Figure 1d), evidenced by the shift to the left of the 2*θ* diffraction angle. Non-oxidized GNP powders presented sharp and strong diffraction peaks (002 lattice) at 2*θ* = 26.5°, indicative of their ordered, stacked structure and reduced interlayer spacing between sheets (d = 3.4 nm). Oxidized powders exhibited a lower diffraction angle (001) at 2*θ* =~11.0°, corresponding to an increase in the interlayer spacing between sheets (*d* = ~8.0 nm), associated with the presence of oxygen-containing groups.

**Figure 2:**
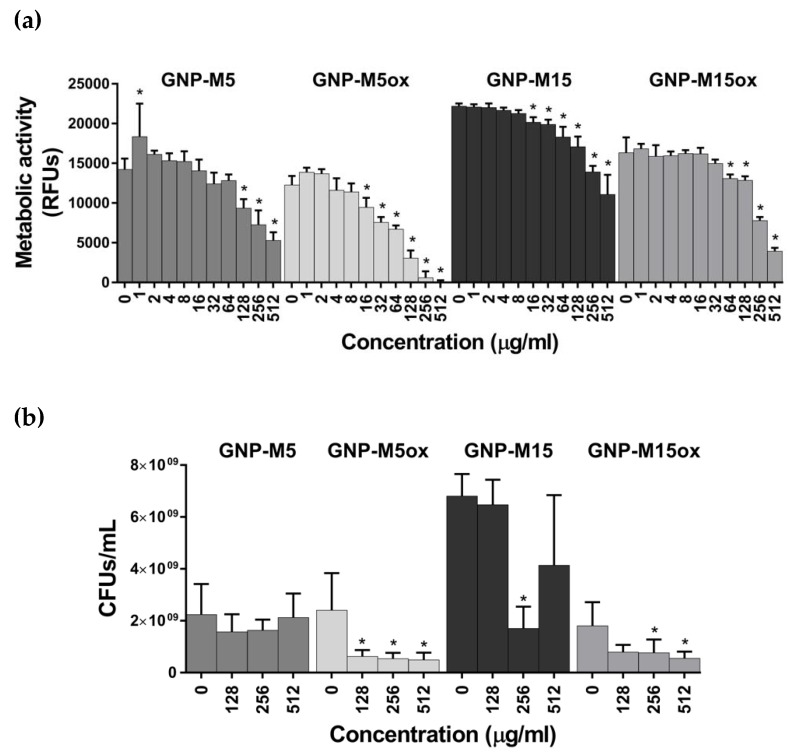
Antibacterial activity of GNP dispersions: (**a**) metabolic activity determined by resazurin assay and (**b**) bacteria vibility of *S. epidermidis* determined by viable cell count after 24 h incubation with GNP-M5, GNP-M5ox, GNP-M15 and GNP-M15ox aqueous dispersions. * Statistically significantly different from control (one-way ANOVA (A) and Kruskal–Wallis (B); *p* ≤ 0.05).

Antibacterial assessment revealed a decrease in metabolic activity with increasing GNP concentration, reaching reductions of 63%, 100%, 50% and 76% for the 512 μg/mL dispersions of GNP-M5, GNP-M5ox, GNP-M15 and GNP-M15ox, respectively (Figure 2a). Bacteria viability decreased particularly when in contact with oxidized materials, namely with 74% and 57% killing when in contact with 128 µg/mL of GNP-M5ox and 256 µg/mL of GNP-M15ox, respectively; the highest decrease of bacteria viability of 82% and 69% occurred with 512 μg/mL dispersions of GNP-M5ox and GNP-M15ox, respectively (Figure 2b). In the GNP-M5 dispersions, bacteria were metabolically less active (Figure 2a) but not dead since growth was observed in the viable cell counting method (Figure 2b).

When comparing GNP with different sizes, GNP-M5 led to a higher decrease on the metabolic activity than GNP-M15 (Figure 2a); the same tendency occurred for the GNP-M5ox and GNP-M15ox. This suggests that smaller particles (5 µm) have higher bacterial inhibitory effect. When comparing particles with different oxidation degree, higher oxidation led to a higher reduction in bacteria viability, leading to greater CFUs/mL reduction (Figure 2b). Bacteria were metabolically inactive but still viable when cultured with non-oxidized GNP and, on the other hand, metabolically inactive and dead when cultured with oxidized GNP. As powders in suspension, GNP-M5ox showed to possess stronger antibacterial activity than the other materials, expressed by a significant decrease of both metabolic activity (100%) and cultivable bacteria counting (82%) when 512 µg/mL were used. The impact of GNP lateral size was also explored on Rago et al. work [65], with lower thickness and smaller size GNP exhibiting stronger antibacterial activity towards *Streptococcus mutans* (500 μg/mL) than larger and thicker ones, with reductions of cell viability of 80% and 65%, respectively. However, the effect of GNP oxidation, namely comparison between different GNP (FLG or MLG) and GNPox alone on the antibacterial properties has, to our knowledge, never been reported. Most of the studies explore the relation between GO and reduced graphene oxide (rGO) in dispersion [29,66,67,68,69,70]—*E. coli* expressed a viability loss up to 98.5% when treated with 85 µg/mL GO for 2 h, higher than with rGO (≈90%) [29]; when increasing the concentration of GO and rGO to 150 µg/mL, the same tendency was maintained with *E. coli* presenting a viability loss of 87% and 81%, respectively [69]; increasing even more the concentration (250 μg/mL) and extending the period of incubation (18 h) with GO and rGO led to complete inhibition of the growth of *Listeria monocytogenes* and *Salmonella* [70]. Liu and co-workers [66] corroborated the higher antibacterial activity of smaller and more oxidized materials, demonstrating an increased antimicrobial potency for GO (0.31 μm) > rGO (2.75 μm) > graphite (6.87 μm) > graphite oxide (6.28 μm) dispersions (40 µg/mL) expressed by 70%, 46%, 26% and 15% of *E. coli* viability loss after 2 h. GNP and GNPox behaved similarly in our study, with smaller and oxidized particles having higher antibacterial potential.

### 3.2. PU/GNP Composites

**Figure 3:**
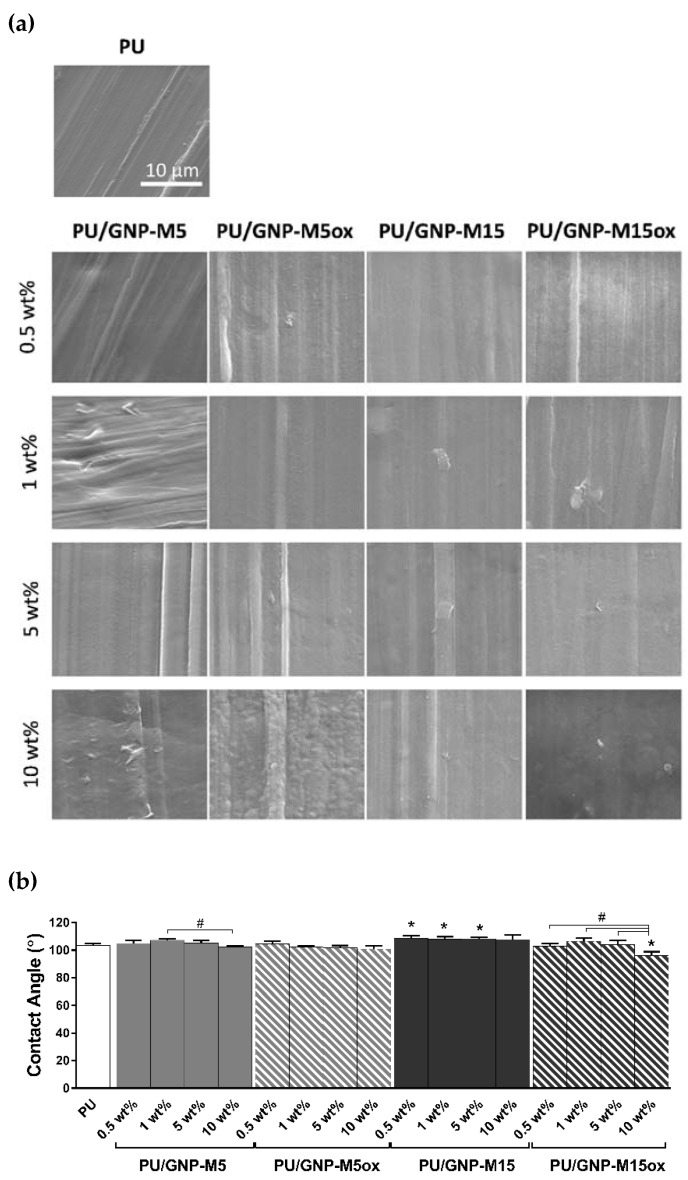
Characterization of polyurethane (PU)/GNP composites: (**a**) surface topography and (**b**) wettability of PU and PU/GNP composites with different GNP content produced by melt-blending. Images were obtained by SEM (scale bar = 10 µm) and wettability determined by water optical contact angle measurements. * and # statistically significantly different from control (PU) and differences between samples, respectively (one-way ANOVA; *p* ≤ 0.05).

GNP incorporation in the PU matrix was visually confirmed through an obvious change in color—from transparent PU into black opaque composites—even for the lower amount of GNP (0.5 wt%), due to the high specific surface area of the nanoplatelets (Figure S1). The effective incorporation of GNP in the PU matrix was corroborated by SEM of the fracture surfaces (Figure S2): in comparison with neat PU that has a smooth cross-section and surface, PU/GNP composites presented a rougher surface with apparent irregular stripes, as also reported by Tang and co-workers upon GO incorporation [47]. A homogenous dispersion of fillers was achieved, with an increase of GNP concentration leading to more platelets with sharp edges in the PU matrix and to a rougher fracture. Composites containing oxidized GNP (GNP-M5ox and GNP-M15ox) presented platelets with wrinkled appearance and folded edges, as observed in the oxidized GNP powders. A good adhesion at the interface between the GNP and the PU was observed, even without using any compatibilizers, as observed by others [56,58]; some aggregates could be found in composites containing the highest GNP amount (10 wt%), particularly when larger and oxidized GNP were used. However, surface morphology of the composites was rather similar to the neat PU surface, with very few platelets being exposed—may be partially or completely covered by a layer of polymer—and usually found in a planar orientation, independently of the type and loading of the GNP incorporated (Figure 3a).

Regarding surface wettability, all composite samples presented similar water contact angles, associated with the poor GNP exposure at the surface, and higher than 90°, independently on the oxidation degree. Only when incorporating GNP with larger lateral size significant differences from the neat PU were observed: incorporation of GNP-M15 (0.5, 1 and 5 wt%) contributed to a significant increase in PU hydrophobicity (angle increase around 4–5°) whereas GNP-M15ox (10 wt%) incorporation led to a significant decrease in PU hydrophobicity (angle decrease of 7°) (Figure 3b).

**Figure 4:**
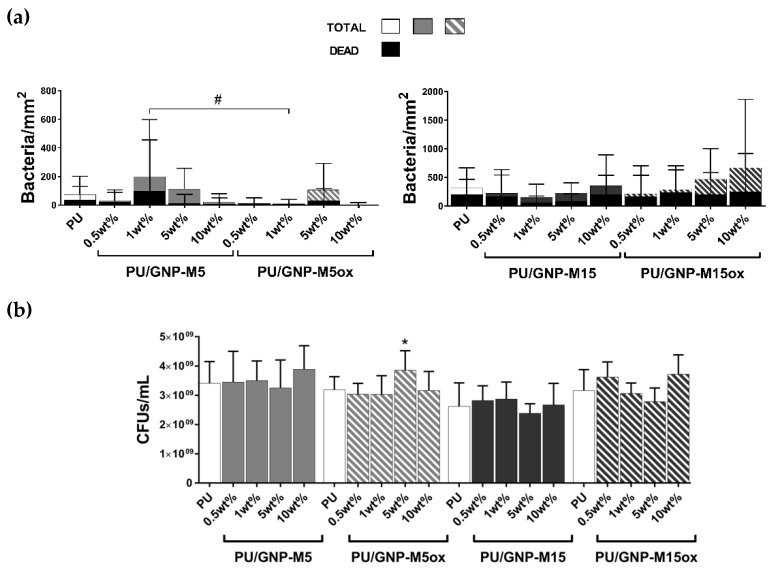
Antibacterial activity of PU/GNP composites after 24 h incubation with *S. epidermidis*: (**a**) total and dead adherent bacteria per mm^2^ determined after total/dead staining and (**b**) planktonic viable bacteria collected in supernatant determined through viable cell counting. * and # statistically significantly different from PU (control) and differences between samples, respectively (Kruskal–Wallis (A) and one-way ANOVA (B); *p* ≤ 0.05).

The antibacterial potential of these composites upon contact with *S. epidermidis* was low, both towards adherent and planktonic bacteria: no statistically significant differences were observed in the number of total or dead adherent bacteria compared to neat PU for any of the composites (Figure 4 and Figure S3) nor in the bacteria viability and metabolic activity of planktonic bacteria (Figure 4b and Figure S4). Common LIVE/DEAD kits could not be used due to the presence of green fluorescent stains: initial tests demonstrated PU autofluorescence in this channel. Incorporation of the different GNP onto PU neither decreased the bacterial adhesion nor increased the bactericidal activity of PU.

Melt-blending represents an economically attractive and industrially scalable method for efficiently dispersing nanofillers in polymers, being compatible with the manufacturing processes for catheter production [9,71]. Besides, this method does not involve the use of solvents, representing an environmentally friendly method that avoids concerns with human health during processing and with toxicity of remaining solvent residues [72]. However, although melt-blending allowed GNP to be effectively dispersed within the polymer matrix, surface characterization showed that this was not a good method to expose GNP at the surface and, consequently, favor GNP contact with bacteria; this can justify the lack of antibacterial properties of the composites.

### 3.3. PU/GNP Coatings

**Figure 5:**
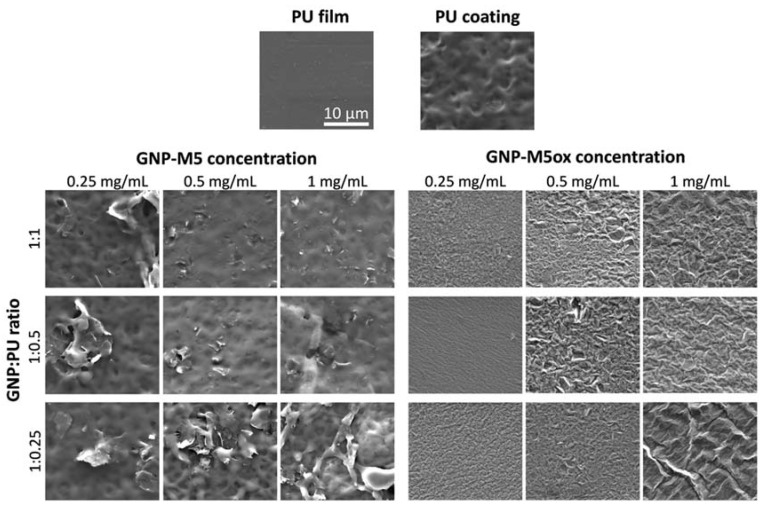

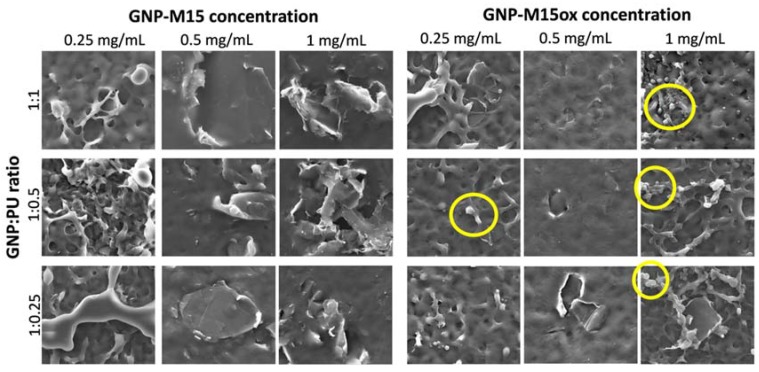
Characterization of PU/GNP coatings: surface topography of PU film (substrate), PU coating (PU coating on PU substrate) and PU/GNP coatings with different GNP concentration and GNP:PU ratio produced by dip coating with different GNP concentration and GNP to PU weight ratio. Images were obtained by SEM (scale bar = 10 µm); yellow circles indicate spherical agglomerates observed in the surfaces.

Composite dispersions containing PU and different GNP—GNP-M5, GNP-M5ox, GNP-M15 and GNP-M15ox—were applied as a coating onto PU substrates by dip coating. GNP concentration was varied (from 0.25 to 1 mg/mL) to understand if there was a direct relation between GNP concentration and exposure at the surface whereas the ratio of GNP to PU was increased (from 1:1 to 1:0.25) to evaluate if the PU content could be lowered and still ensure GNP adherence to the substrate. A change in color was visible for all samples, ranging from transparent PU to black or brownish color when GNP or oxidized GNP were incorporated, respectively; increasingly darker appearance occurred as GNP concentration increased (Figure S5). All coatings produced from different batches were homogenous. Comparing to composites obtained by melt-blending (Figure 3a), these PU/GNP coatings presented a mesh-like appearance (attributable to solvent evaporation and consequent PU precipitation) and much higher amounts of GNP platelets exposed at the surface (Figure 5). Particularly in coatings with non-oxidized GNP (GNP-M5 and GNP-M15) many platelets with sharp edges were bursting from the coating surface and at different orientations, although some of these platelets were also partially or completely covered with polymer; especially in coatings with higher GNP-M15 loading (1 mg/mL) some platelet aggregation took place. When decreasing the PU content (GNP:PU ratio increasing from 1:1 to 1:0.25), the surface acquired a more mesh-like and irregular topography with platelets less covered by polymer as particularly observed with GNP-M5 incorporation: the lower amount of PU present in the solution, although not enough for complete uniform surface coverage, allowed platelets to stick to the PU film substrate. As expected, the number of platelets and aggregates observed at the surface increased with GNP concentration. Individualized platelet-like structures were scarcely seen in the coatings with oxidized GNP (Figure 5): coatings with GNP-M5ox presented homogeneous creased surfaces composed of apparently fused wrinkled GNP-M5ox sheets with some protruding edges; coatings with GNP-M15ox also had areas with a similar wrinkled topography, but the presence of spherical wrinkled agglomerates was also visible. The wrinkled topography and formation of spherical structures indicates strong polymer–GNPox interactions [61], behavior related to the morphology and surface chemistry of the oxidized GNP where oxygen-containing groups present at the surface may have formed hydrogen bonds with the N-H groups present in the PU molecule, leading to strong interfacial interactions [45,73]. In the liquid-phase mixing method used, when PU was mixed with oxidized GNP sheets, they preferentially stuck together, causing agglomerated oxidized GNP/PU structures to be formed upon solvent evaporation [45]. Differences in lateral size can also contribute to the different morphology observed, both with non-oxidized and oxidized platelets, where the greater the sheet size the more irregular the surface, due to the aggregation of the GNP sheets and interaction with the polymer. Similar surface topographies were obtained by our group by dip or spray coating silicone films, obtaining exposed platelets and aggregates or creased structures when using either GNP-M5 or GNP-M5ox [50]. Flat coatings without sharp edges, identical to the coatings containing GNP-M5ox obtained here were also reported by others using GO [20]. Thampi et al. also observed wrinkled topography when spraying GO onto electrospun polycarbonate urethane (PCU) membranes [74]. Overall, coatings with GNP:PU ratio of 1:0.5, and specially with GNP concentration of 0.5 mg/mL, seemed to present more uniform and thus more promising surfaces. 

GNP coatings’ adherence to the PU substrate was confirmed by the rubbing test with no GNP detachment being observed for all coatings, except for the coatings containing higher GNP content (1 mg/mL), with the resistance of the coating decreasing as the GNP:PU ratio increased. This result is not surprising as PU in solution works as binder between GNP and the PU film surface. 

**Figure 6:**
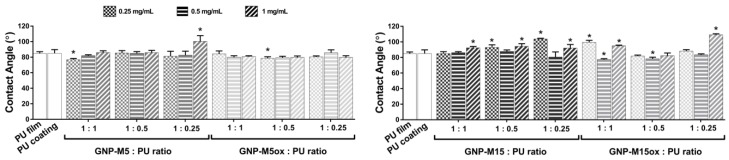
Characterization of PU/GNP coatings: surface wettability of PU (control) and PU/GNP coatings with different GNP concentration and GNP:PU ratio, obtained by water optical contact angle measurements. * statistically significantly different from control (PU) (one-way ANOVA; *p* ≤ 0.05).

Regarding surface wettability, both PU film (substrate) and PU coating on PU film exhibited a water contact angle of 85° (Figure 6), less hydrophobic than PU produced by melting and injection (103°) (Figure 3b). Some variability regarding PU Tecothane surface wettability has been found throughout the literature—surfaces produced by solvent casting presented angles ranging from 90° to 73° [75,76], with the difference between studies relying on the solvent used to dissolve PU: DMF and THF, respectively; surfaces produced by solvent polymerization and curing presented a contact angle of 103° [77]—suggesting that not only the production method impacts surface wettability but also the materials interacting with the polymer. When producing the coating with PU/GNP-M5 composite, wettability change was only detected for the formulation with 0.25 mg/mL GNP and 1:1 GNP:PU ratio and with 1 mg/mL and 1:0.25 GNP:PU ratio, with the water contact angle of PU decreasing to 77° and increasing to about 100°, respectively. As observed in composites, when producing coating with larger GNP-M15 platelets, the hydrophobicity increased significantly in many samples and more evidently for 0.25 mg/mL GNP and GNP:PU ratio of 1:0.25, with an increase of the angle to 104°. Increasing the GNP-M15 concentration (1mg/mL) led to a significant increase in the hydrophobicity (from 85° (neat PU) to 93°), which is justified by the presence of more platelets and aggregates exposed at the surface, forming hydrophobic sites. Similar results have been reported for poly(vinyl acetate) (PVAc) with incorporation of 0.1 wt% GNP [78]. The impact of oxidation degree was less notorious independently of the size of the platelets (PU/GNP-M5ox and PU/GNP-M15ox), being roughly the same for all samples with no obvious tendency observed, which correlates with the similar topography observed for all these surfaces. There was however a decrease of the angle (to 78°), and thus an increase of the hydrophilicity, for 0.5 mg/mL GNP-M15ox and GNP:PU ratio of 1:1 and 1:0.5, suggesting that surface topography might play a role in wettability, with these surfaces being apparently smoother than the remaining conditions. When increasing the concentration (from 0.25 mg/mL to 1 mg/mL) for the same GNP:PU ratio (1:1), surface hydrophobicity decreased (from 99° to 95°), even though not significantly. At the highest concentration of GNP-M15ox, when reducing the amount of PU, an increase in hydrophobicity was observed (to 109°), even though more platelets are exposed; once again, surface topography may overrule chemistry on the effect of GNP exposure. It would be expected that incorporation of oxidized GNP increased the hydrophilicity due to the hydrogen bond interactions between the oxygen-containing groups present in GNP and water. Our group has previously reported a water contact angle decrease of ~9° on PLA/GO films compared to pristine PLA films [79]; others have also shown that incorporation of GO into PU decreased the water contact angle [80]. However, wettability is affected not only by chemical composition but also by surface roughness. Interestingly, for the coatings there seems to be a relation between the extent of irregularities at the surface, more randomized mesh and bigger defects, and the higher changes of water contact angle. In the composites, an increase of the hydrophilicity was also only observed with the highest GNP-M15ox loading (10 wt%), with surface roughness being similar between conditions.

**Figure 7:**
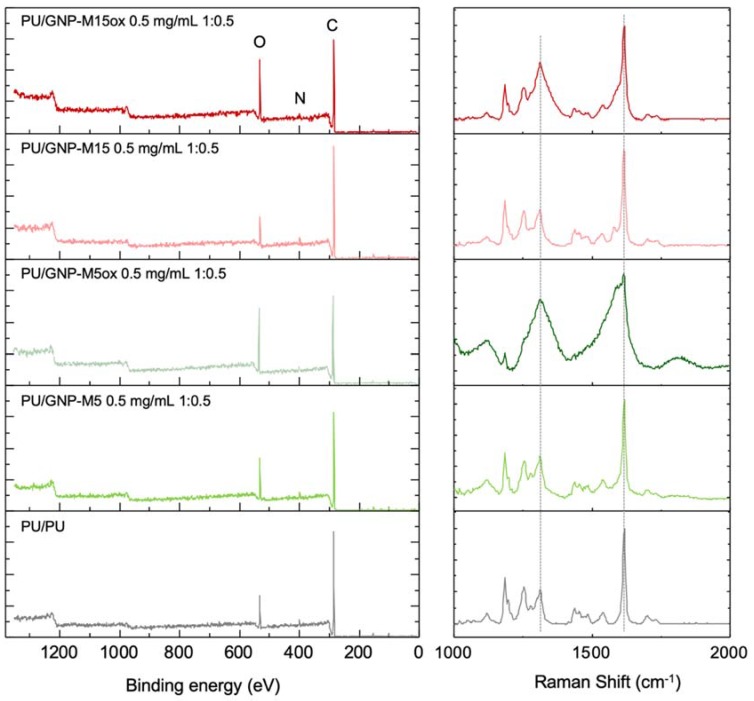
Characterization of surface chemistry of PU/PU and PU/GNP coatings with intermediate GNP concentration (0.5 mg/mL) and intermediate GNP:PU ratio (1:0.5): XPS survey spectra (left panel) and Raman spectra (right panel).

Surface chemistry of the PU/GNP coatings was further explored using XPS and Raman spectroscopy, being PU/GNP coatings with intermediate GNP concentration (0.5 mg/mL) and intermediate GNP:PU ratio (1:0.5) selected for this analysis (Figure 7). XPS spectra showed that the PU/PU coating is mainly constituted by carbon (87.8%), oxygen (9.4%) and nitrogen (2.8%), as expected. Coating the surface with PU/GNP-M5ox and PU/GNP-M15ox increased the amount of oxygen to ~16%, as noticed by the increase of the oxygen peak in the survey, highlighting the presence of the oxygen-containing groups from the oxidized GNP in the upper 10 nm of the coatings. Raman spectra showed the characteristic bands of PU in all the analyzed surfaces, demonstrating the PU presence in all coatings. The presence of GNP-M5 and GNP-M15 in the coatings could not be confirmed since the strong, sharp G band (~1585 cm^−1^ [81]) typical from these materials overlapped with the C=C bond from PU at 1617 cm^-1^. For PU/GNPox, the presence of strong and broad D (~1310 cm^−1^) and G (~1615 cm^−1^) bands, characteristic of oxidized forms of graphene can be easily identified. G peak corresponds to the bond stretching of sp^2^ carbon atoms, while the D band is associated with the structural defects present in the GNPox rings introduced due to their oxidation process [82]. Therefore, XPS and Raman spectra confirmed the presence of both PU and GNP or GNPox at the surface of the composite coating, presenting different surface chemistry.

**Figure 8:**
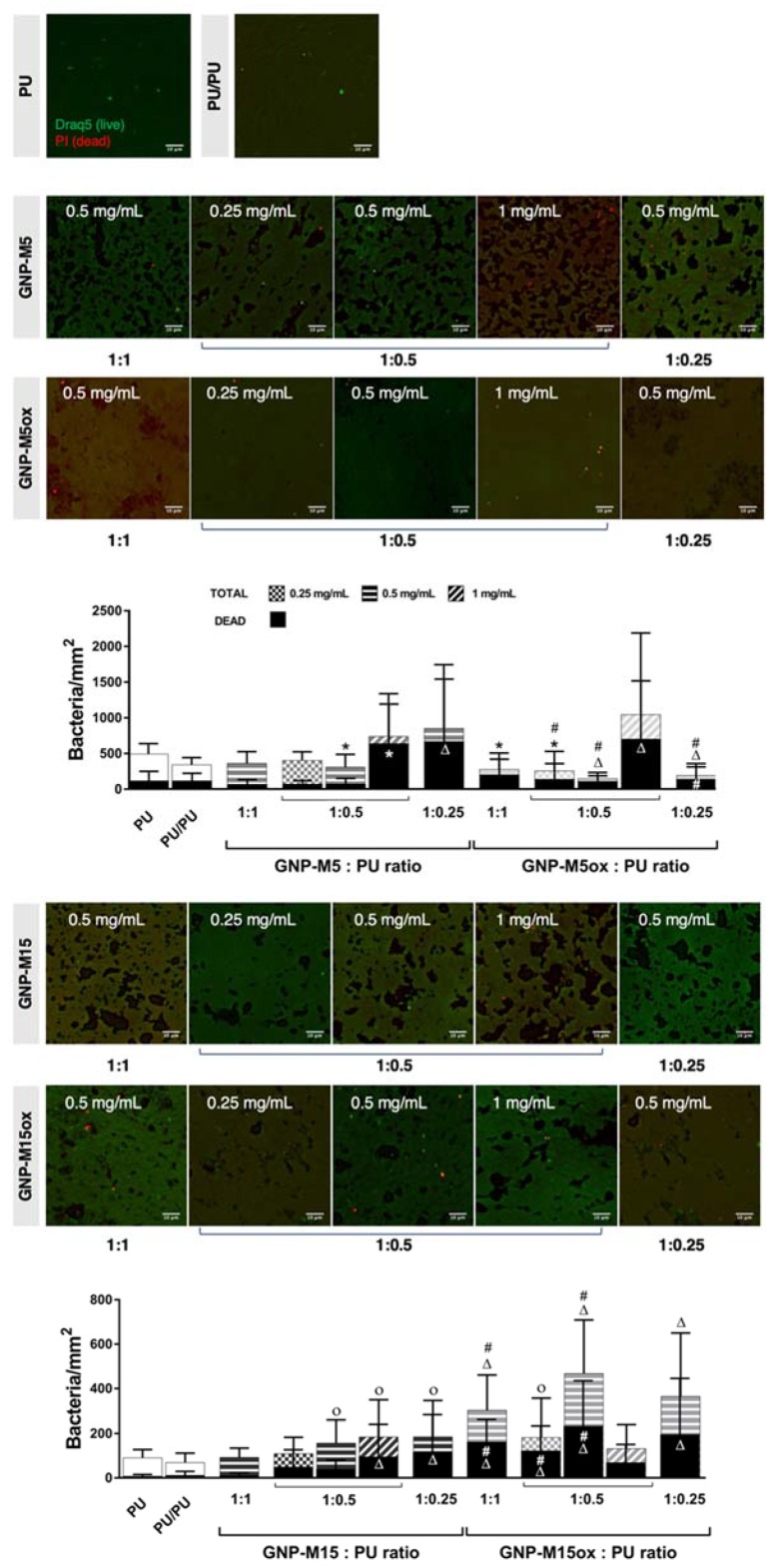
Antibacterial activity of PU/GNP coatings with different GNP concentration and GNP:PU ratio towards adherent *S. epidermidis* after 24 h incubation. Images of live/dead bacteria staining (Draq5/PI) (scale bar = 10 µm) and total and dead adherent bacteria per mm^2^ determined from total/dead staining. *, o, *∆* and # statistically significantly different from control PU film (PU), from PU coating on PU (PU/PU), from both and differences between the correspondent non-oxidized samples, respectively (Kruskal–Wallis; *p* ≤ 0.05).

In order to study the impact of either GNP concentration and PU:GNP ratio on the antibacterial potential towards *S. epidermidis*, one of the variables was maintained while varying the other: GNP concentration of 0.5 mg/mL was fixed while varying PU:GNP ratio (1:1, 1:0.5 and 1:0.25) and the other way around, fixing the PU:GNP ratio of 1:0.5 and varying GNP concentration (0.25, 0.5 and 1 mg/mL). Regarding total adherent bacteria (Figure 8), there was a significant decrease of 37% when using GNP-M5 at 0.5 mg/mL and GNP:PU ratio of 1:0.5 when comparing to bare PU film. Increasing the concentration to 1 mg/mL or the GNP:PU ratio to 1:0.25 caused a significant increase of dead bacteria to 78% and 85%, respectively. These conditions contained higher amounts of sharp graphene nanoplatelets, suggesting the role of these structures on the effective killing of bacteria after contact with the surface. In oxidized samples with the same lateral size (GNP-M5ox) there was a reduction in bacterial adhesion up to 70% (ranging from 44% to 70% in different conditions) while increasing bacterial death (ranging from 53% for the lower concentration and ~70% in all other conditions); the exception was the condition with the highest amount of GNP-M5ox (1 mg/mL), showing high bacterial adhesion, although at the same time high killing ability (around 70%). The wrinkled morphology of the surfaces with GNP-M5ox associated with the oxidation of the surface may be behind this effect, preventing bacteria to adhere in high number while prompting some oxidative stress mechanism. Increasing platelets lateral size (GNP-M15) led to an increase in bacterial adhesion and death (reaching 62% and 71% bacterial death) when comparing to PU, specifically in conditions with lower amount of PU on the composite coating (0.5 mg/mL, ratio of 1:0.25) and with higher amount of GNP (1 mg/mL), suggesting that the exposure of the sharp edges of non-oxidized GNP and associated increase of surface area/roughness promotes bacterial adhesion despite killing them. Contrarily to GNP-M5ox, GNP-M15 oxidation (GNP-M15ox) significantly boosted bacterial adhesion in most of the conditions. The lower amounts of oxygen-containing groups in platelets with higher lateral size seems to prevent the formation of a continuous layer as observed in PU/GNP-M5ox surfaces, resulting in a surface with high roughness exposing both GNPox and PU, which can promote bacterial adhesion. The percentage of dead bacteria was on the other hand significantly higher than in PU and their non-oxidized counterparts, reaching 67% in conditions with lower amount of PU on the composite coating (0.5 mg/mL, ratio of 1:0.25).

Overall, when using non-oxidized GNP-M5 and GNP-M15, the highest bacterial death was observed when higher amounts of GNP are exposed (at the highest GNP:PU ratio of 1:0.25 and 0.5 mg/mL GNP and at the highest GNP concentration of 1 mg/mL). With oxidized GNP, more death was also observed in the coatings with 0.5 mg/mL and 1 mg/mL of GNP-M5ox. Increasing GNP:PU ratio (1:0.25), thus decreasing the amount of PU in the coating formulation, improved the exposure of platelets at the surface, which also seemed less covered by the polymer; this allowed more contact with bacteria. Increasing GNP concentration increased the chances of exposing platelets at the surface and thus the contact with bacteria. This correlates with the literature that states the importance of GBM sheets orientation, exposure and concentration on the antibacterial performance of surfaces integrating GBM [40,83]. Therefore, increasing platelets exposure increased contact with bacteria, which consequently seemed to potentiate the effects of the sharp edges and basal planes of the GNP platelets, either due to disruption of the cell membrane [36,84] or oxidative stress induction [41,85].

**Figure 9:**
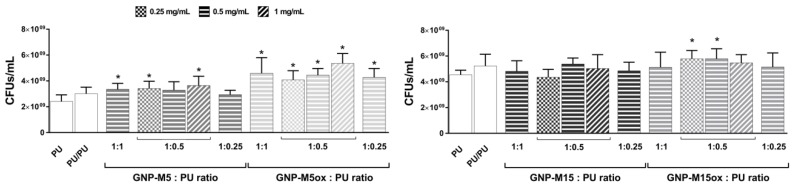
Antibacterial activity of PU/GNP coatings with different GNP concentration and GNP:PU ratio towards planktonic *S. epidermidis* after 24 h incubation determined by viable cell count. *, o, *∆* and # statistically significantly different from control PU film (PU), from PU coating on PU (PU/PU), from both and differences between the correspondent non-oxidized samples, respectively (one-way ANOVA (B); *p* ≤ 0.05).

Concerning planktonic bacteria, in most of the surfaces, neither bacteria viability (Figure 9) nor metabolic activity (Figure S6) decreased. This result was also expected since these surfaces do not act as leaching systems and therefore the effects of GNP will only be exerted against the bacteria in contact with the surface. However, specifically in PU/GNP-M5ox surfaces where bacterial adhesion was reduced, viable bacteria on the supernatant significantly increased when compared to PU film, suggesting that bacteria were impaired to adhere but survived and grew in the supernatant. 

As aforementioned, PU/GNP composites produced by melt-blending lacked GNP exposure at their surface, therefore failing on improving the antibacterial properties of PU. This was overcome through the application of PU/GNP composite as a coating on PU by dip coating, where higher GNP exposure at the surface was achieved, which, in some of the conditions, was translated into a reduction/increase in bacterial adhesion and/or increase in bacterial death. GNP exposure is therefore crucial for the antibacterial activity of the GBM-integrating surfaces, being potentiated when increasing the amount of platelets present and/or reducing the presence of the polymer. This affects the way GBM are deposited and oriented on the surface, thus impacting bacteria-GBM interaction and ultimately the antibacterial activity of the surfaces [40]. This relation of GBM exposure with the antibacterial activity of GBM-containing surfaces has been suggested in other studies, as recently by Cheng et al. [83]. Even though antibacterial activity has been observed both when basal planes [84,85,86] or sharp edges are exposed [87], a direct contact between bacteria and the surface must occur, as reinforced by the absence of antibacterial effects both on the bacteria incubated with the composites and on bacteria collected in the supernatant with both surfaces. 

Apart from GNP exposure, we have also established the influence of oxidation degree by reporting higher antibacterial activity on PU/GNP coatings containing GNPox than on coatings with non-oxidized GNP, as also supported by a study comparing other GBM (namely GO with rGO [29]) and by our previous work with GNP/silicone coatings [50]. Lateral size also played a role, with smaller platelets being more promising regarding inhibition of bacterial adhesion, particularly, when smaller oxidized particles are used, as GNP-M5ox.This was also supported by several works, as by Perreault et al. that demonstrated that smaller GO sheets presented higher antimicrobial effect, attributed to oxidative mechanisms associated with the higher defect density of smaller sheets, which allow more oxygen to be adsorbed on them [41]. Bigger platelets (GNP-M15) led to the presence of topography irregularities, which seem to favor bacteria adherence. At the same time, this induces higher percentage of death. Morphology and topography of the surfaces play therefore a crucial role on the antibacterial potential of the surfaces, while on surfaces containing GNP the action towards bacteria can be attributable to mechanical damages provoked by the sharp edges of the exposed nanoplatelets, on surfaces containing GNPox, particularly GNP-M5ox, the wrinkled topography and the absence of sharp edges indicates oxidative stress induction.

Coatings containing GNP-M5ox revealed to be the most promising by having both antifouling and bactericidal effects towards *S. epidermidis*. Therefore, these coatings are the most suitable for the development of an antimicrobial material.

## 4. Conclusions

This work demonstrates the relevance of GNP oxidation and lateral size on the antimicrobial profile of GNP both as dispersions and surfaces. Oxidized and smaller particles (GNP-M5ox) in suspension induced stronger activity against *S. epidermidis*, decreasing bacteria viability in 74%. The impact of GNP exposure on the surface was stated by comparing surfaces produced by melt blending—bulk PU/GNP composite—and dip coating—PU/GNP composite layer on top of a substrate. Absence of GNP exposure at the surface of PU/GNP composites resulted in the lack of antibacterial effect towards *S. epidermidis*. On the other hand, PU/GNP coatings yielded surfaces with well-adhered and exposed nanoplatelets, revealing an improvement on antibacterial and anti-adhesive properties. Coatings containing smaller platelets (GNP-M5 and GNP-M5ox) caused higher antibacterial activity than coatings with larger platelets (GNP-M15 and GNP-M15ox). Coatings with GNP-M5 showed the highest increase of bacterial death (up to 85%). The most promising surfaces were however the PU/GNP coatings containing GNP-M5ox at a concentration of 0.5 mg/mL and GNP:PU ratio of 1:0.5, which presented both antifouling (70% reduction of bacterial adhesion) and bactericidal activity (70% bacterial death) towards *S. epidermidis* when comparing to bare PU surface.

As such, dip coating technique can be explored as a promising approach to easily transform clinically used catheters into efficient antimicrobial catheters, prolonging their lifetime by reducing the commonly associated risk of infections, thus impacting the life of millions of people. These data should also be considered in the process of designing new antimicrobial polyurethane surfaces in a wide range of applications.

## 5. Patents

PPP 20191000031489. (2019.06.07). ANTIMICROBIAL GRAPHEN COATING. Ana Patrícia Henriques, Inês Borges, Natacha Rosa, Manuel Pestana, Artur Pinto, Fernão Magalhães, Inês Gonçalves.

## Supplementary Materials

The following are available online at https://www.mdpi.com/2079-4991/10/2/349/s1, Figure S1: Images of the PU and PU/GNP composites produced by melt-blending after injection molding, Figure S2: SEM images of PU and PU/GNP composites with different GNP content. Images were taken to the transversal fracture to analyse the composite matrix (scale bar = 10 µm and valid for all images), Figure S3: Representative images of the total/dead bacteria staining (Hoechst/PI) of adherent *S. epidermidis* after 24 h incubation on PU/GNP composites (scale bar = 20 μm). Due to the lower number of bacteria per mm^2^ found at the surfaces and the small area of each field, no or very few bacteria are found in the fields, being these images representative of the total number of bacteria presented in Figure 4a, Figure S4: Metabolic activity of *S. epidermidis* planktonic bacteria after 24 h incubation on PU/GNP composites, Figure S5: Images of the PU film produced by casting, PU coating on PU film and PU/GNP coatings on PU film, Figure S6: Metabolic activity of *S. epidermidis* planktonic bacteria after 24 h incubation on PU/GNP coatings.
