# Erratum: Borges, I., et al. Exposure of Smaller and Oxidized Graphene on Polyurethane Surface Improves its Antimicrobial Performance. *Nanomaterials* 2020, *10*, 349

**DOI:** 10.3390/nano10081470

**Published:** 2020-07-27

**Authors:** Inês Borges, Patrícia C. Henriques, Rita. N. Gomes, Artur M. Pinto, Manuel Pestana, Fernão D. Magalhães, Inês C. Gonçalves

**Affiliations:** 1 i3S—Instituto de Investigação e Inovação em Saúde, Universidade do Porto, Rua Alfredo Allen, 208, 4200-135 Porto, Portugal; ines.s.borges25@gmail.com (I.B.); ap.henriques@ineb.up.pt (P.C.H.); argomes@i3s.up.pt (R.N.G.); arturp@i3s.up.pt (A.M.P.); mvasconcelos@chsj.min-saude.pt (M.P.); 2 INEB—Instituto de Engenharia Biomédica, Universidade do Porto, Rua Alfredo Allen, 208, 4200-135 Porto, Portugal; 3 FEUP—Faculdade de Engenharia, Departamento de Engenharia Metalúrgica e de Materiais, Universidade do Porto, Rua Alfredo Allen, 208, 4200-135 Porto, Portugal; fdmagalh@fe.up.pt; 4 LEPABE, Faculdade de Engenharia, Universidade do Porto, Rua Dr. Roberto Frias, 4200-465 Porto, Portugal; 5 Department of Nephrology, São João Hospital Center, EPE, Alameda Prof. Hernâni Monteiro, 4200-319 Porto, Portugal; 6 Department of Medicine, Faculty of Medicine, University of Porto, Alameda Prof. Hernâni Monteiro, 4200-319 Porto, Portugal

The authors wish to make the following corrections to this paper [1]:

**Funding:** This research was funded by Fundação para a Ciência e a Tecnologia (FCT) and Fundo Europeu de Desenvolvimento Regional (FEDER) for Projects POCI-01-0145-FEDER-006939, POCI-01-0145-FEDER-007274, PTDC/CTM-BIO/4033/2014, and NORTE-01-0145-FEDER-000012, and projects UID/BIM/04293/2019—i3S and UIDB/00511/2020—LEPABE, funded by national funds through FCT/MCTES (PIDDAC).
