# Supplementary material for: Exposure of Smaller and Oxidized Graphene on Polyurethane Surface Improves its Antimicrobial Performance
Source: Nanomaterials (Basel). 2020 Feb 18;10(2):349. doi: 10.3390/nano10020349 (PMC7075169; doi:10.3390/nano10020349)
Supplement: Supplementary file 1 [file nanomaterials-10-00349-s001.pdf]

**Supplementary Material**

# Exposure of smaller and oxidized graphene on polyurethane surface improves its antimicrobial performance

Inês Borges [a,b,#], Patrícia C. Henriques [a,b,c,d,#], Rita. N. Gomes [a,b], Artur M. Pinto [a,b,d], Manuel Pestana [a,b,e,f], Fernão D. Magalhães [d], Inês C. Gonçalves [a,b,*]

[a] i3S - Instituto de Investigação e Inovação em Saúde, Universidade do Porto, Rua Alfredo Allen, 208, 4200-135 Porto, Portugal

[b] INEB - Instituto de Engenharia Biomédica, Universidade do Porto, Rua Alfredo Allen, 208, 4200-135 Porto, Portugal

[c] FEUP – Faculdade de Engenharia, Departamento de Engenharia Metalúrgica e de Materiais, Universidade do Porto, Rua Alfredo Allen, 208, 4200-135 Porto, Portugal

[d] LEPABE, Faculdade de Engenharia, Universidade do Porto, Rua Dr. Roberto Frias, 4200-465 Porto, Portugal

[e] Department of Nephrology, São João Hospital Center, EPE, Alameda Prof. Hernâni Monteiro, 4200-319 Porto, Portugal

[f] Department of Medicine, Faculty of Medicine, University of Porto, Alameda Prof. Hernâni Monteiro, 4200-319 Porto, Portugal

[#] equal contribution

[*] Corresponding author. Tel.: +351 220408800; Email: icastro@ineb.up.pt (Inês C. Gonçalves)

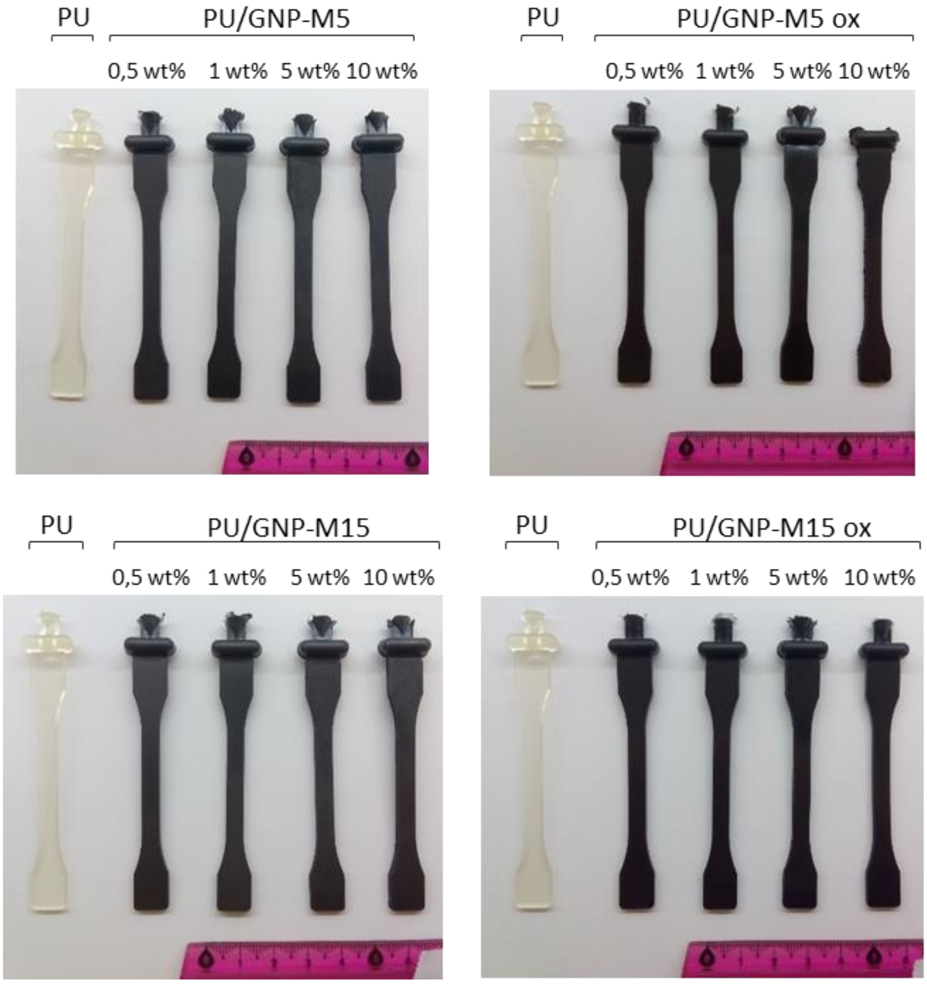


**Figure S1.** Images of the PU and PU/GNP composites produced by melt-blending after injection molding.

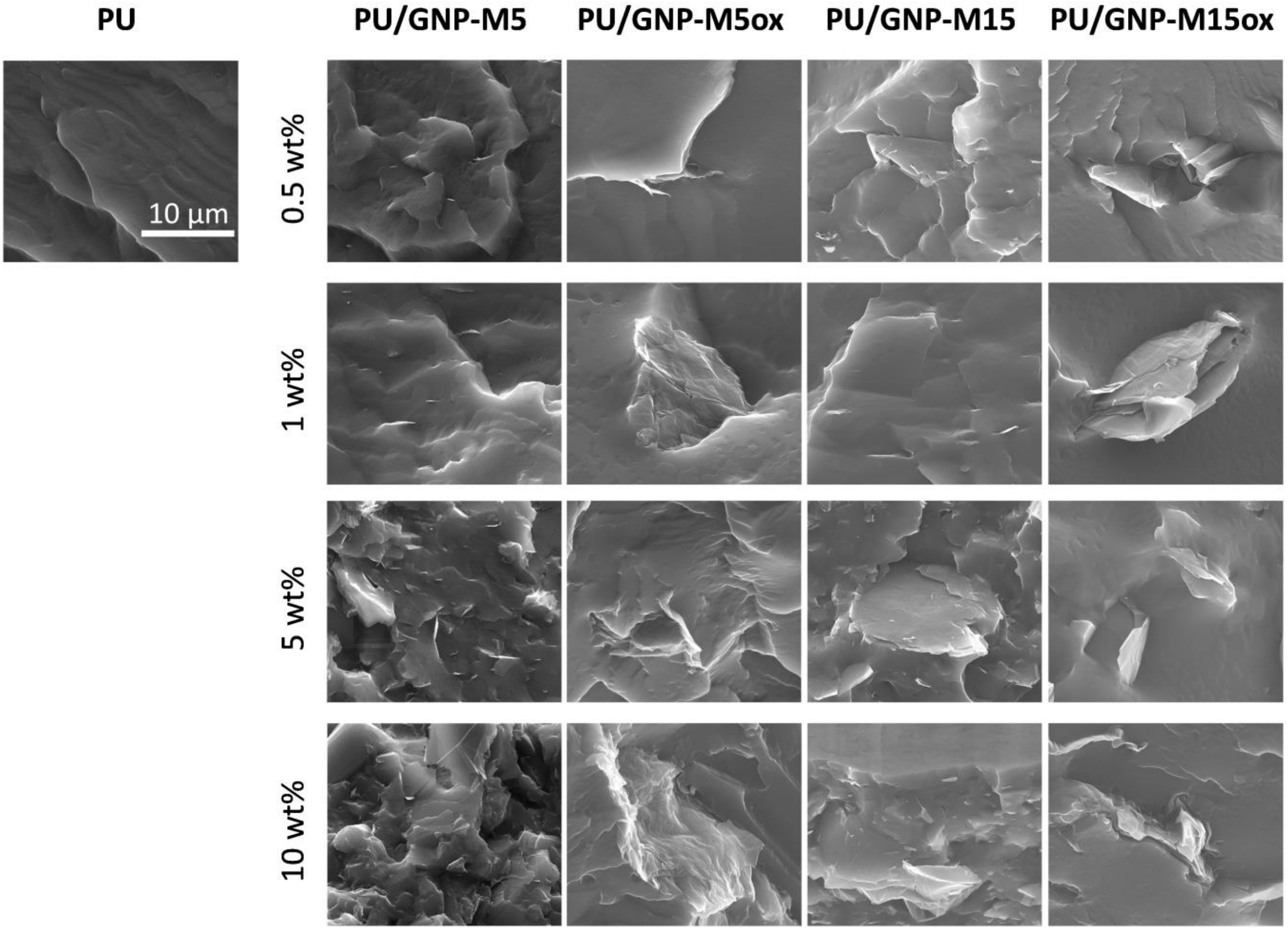


**Figure S2.** SEM images of PU and PU/GNP composites with different GNP content. Images were taken to the transversal fracture to analyse the composite matrix (scale bar = 10 μm and valid for all images).

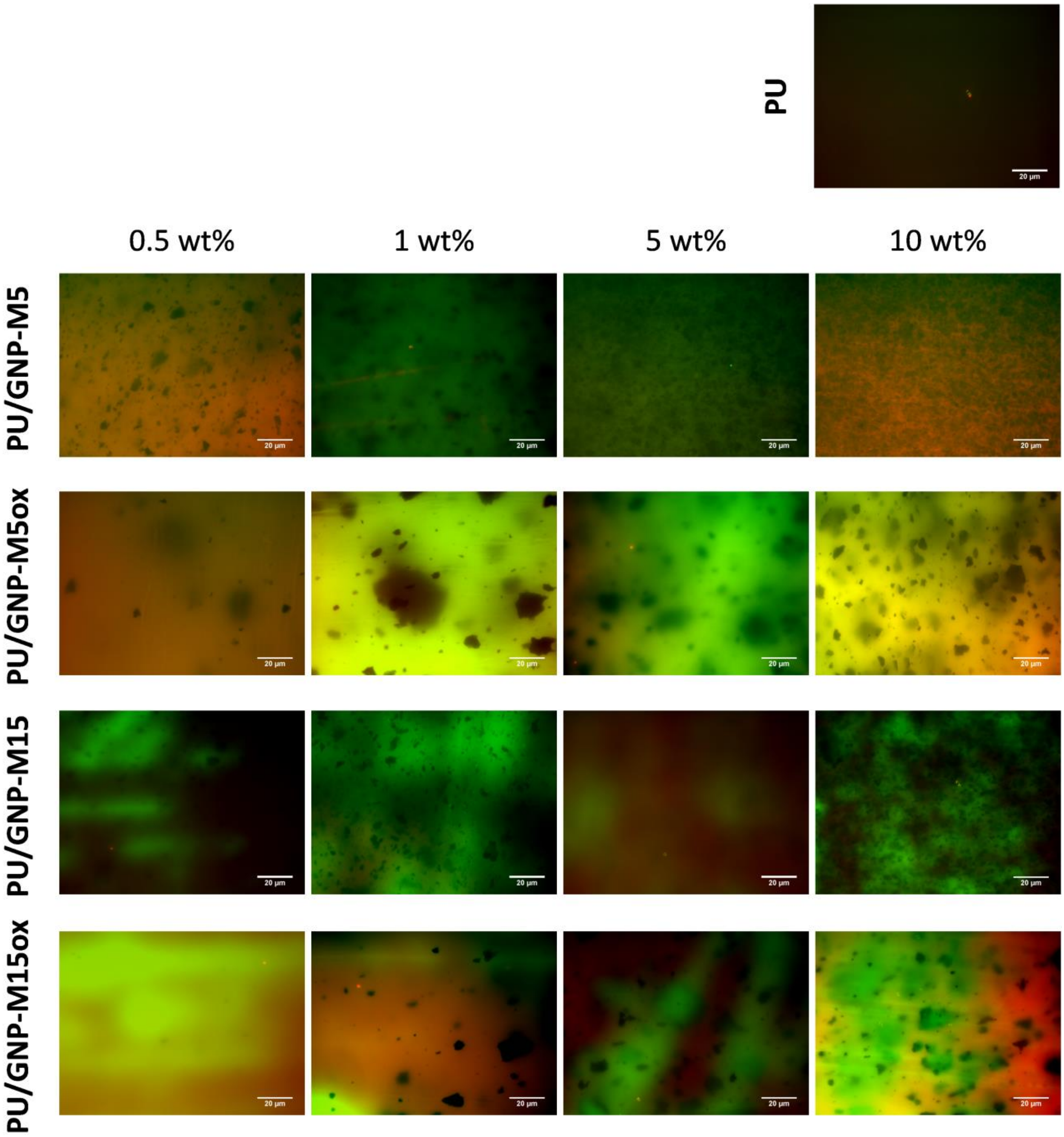


**Figure S3.** Representative images of the total/dead bacteria staining (Hoechst/PI) of adherent *S. epidermidis* after 24 h incubation on PU/GNP composites (scale bar = 20 μm). Due to the lower number of bacteria per $mm^2$ found at the surfaces and the small area of each field, no or very few bacteria are found in the fields, being these images representative of the total number of bacteria presented in Figure 4A.

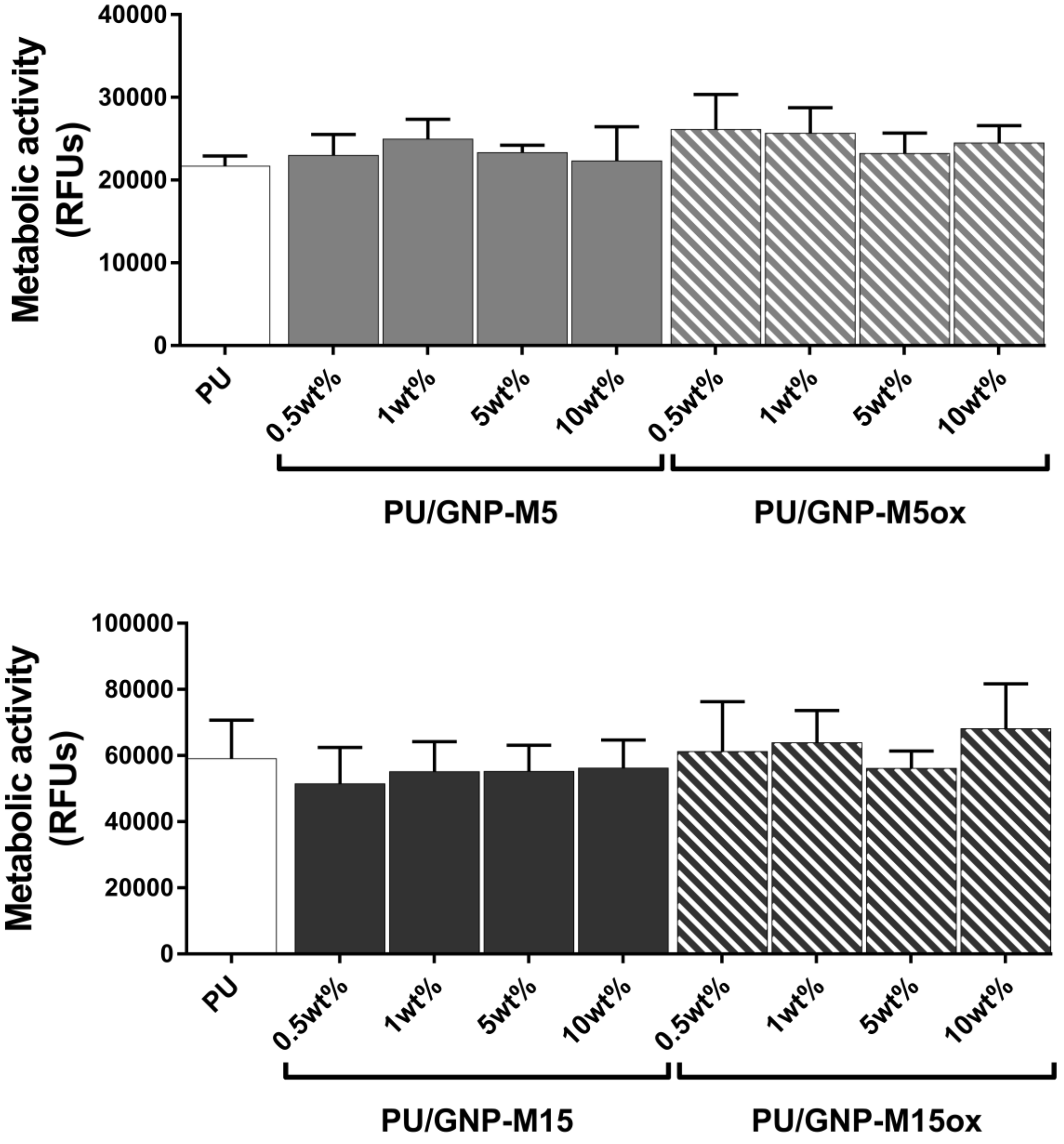


**Figure S4.** Metabolic activity of *S. epidermidis* planktonic bacteria after 24 h incubation on PU/GNP composites.

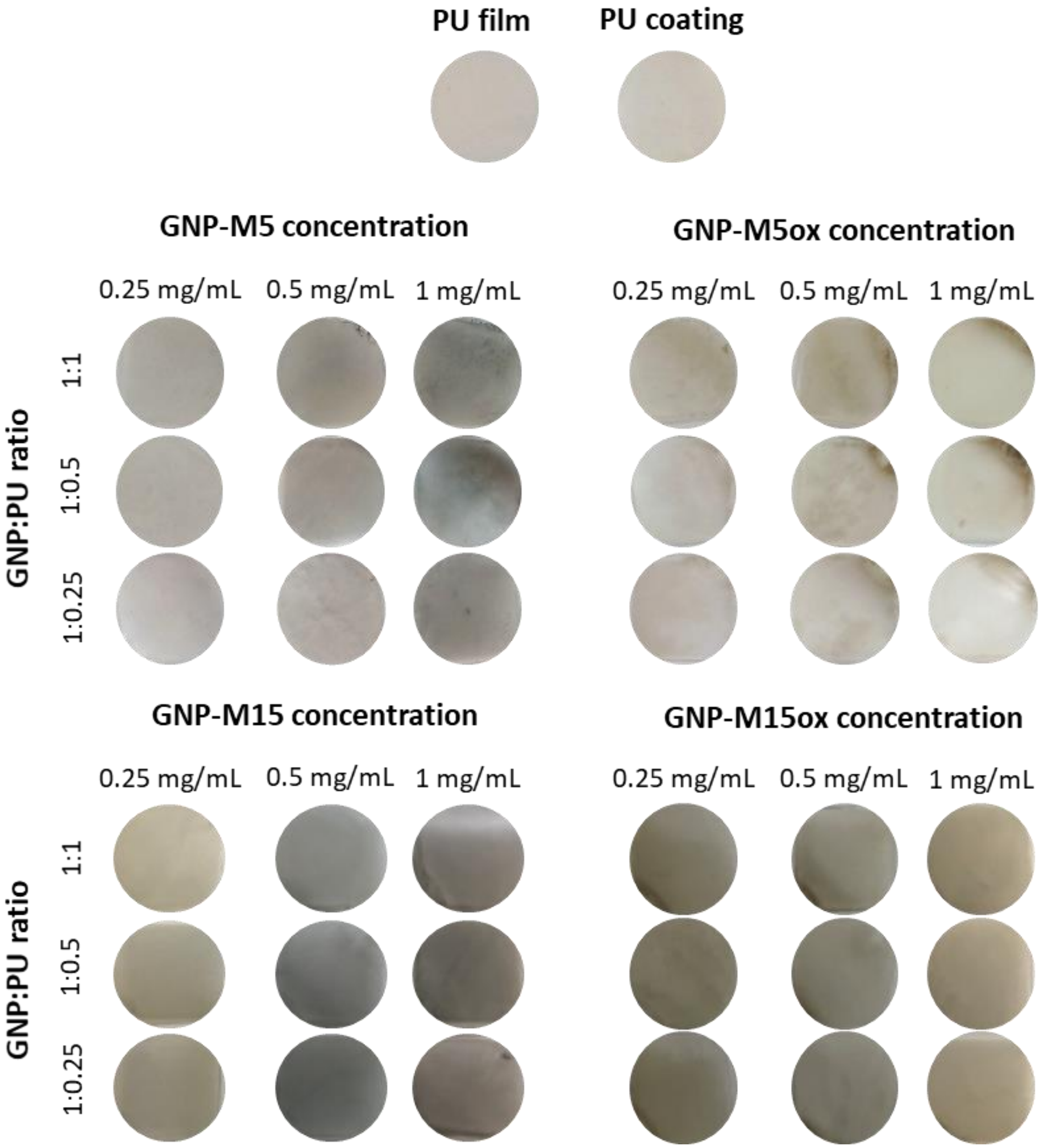


**Figure S5.** Images of the PU film produced by casting, PU coating on PU film and PU/GNP coatings on PU film.

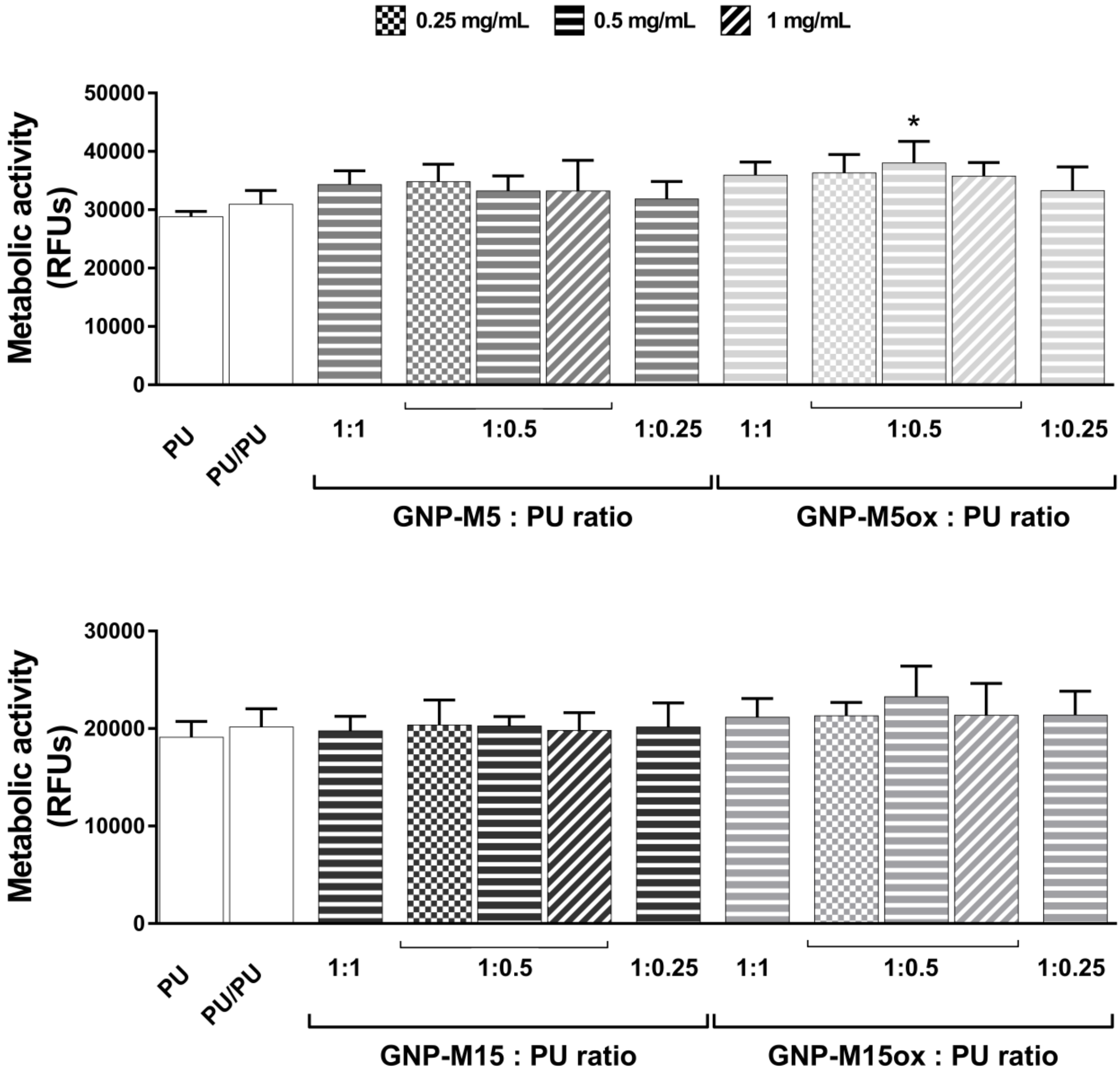


**Figure S6.** Metabolic activity of *S. epidermidis* planktonic bacteria after 24 h incubation on PU/GNP coatings.
